# The Efficacy of Vitamin D Supplementation in Patients With Alzheimer's Disease in Preventing Cognitive Decline: A Systematic Review

**DOI:** 10.7759/cureus.31710

**Published:** 2022-11-20

**Authors:** Mohana Chakkera, Niriksha Ravi, Rajita Ramaraju, Aastha Vats, Athira R Nair, Atithi K Bandhu, Divya Koirala, Manoj R Pallapothu, Maria G Quintana Mariñez, Safeera Khan

**Affiliations:** 1 Internal Medicine, California Institute of Behavioral Neurosciences and Psychology, Fairfield, USA

**Keywords:** cognitive disorders, dementia, cognitive decline, alzheimer's disease, vitamin d

## Abstract

To evaluate the role of vitamin D supplementation in preventing cognitive decline in patients with Alzheimer's disease (AD), five databases such as PubMed, PubMed Central (PMC), Medical Literature Analysis and Retrieval System Online (MEDLINE), ScienceDirect, and Google Scholar were searched for articles relevant to the research question with filters such as English and human studies from 2011 to 2022. Two investigators extracted the data and assessed the quality of the study using the predefined criteria. We identified 24 relevant articles after the critical screening. There were five randomized controlled trials (RCTs), two observational studies, two systematic reviews and meta-analyses, one pilot study, and 14 review articles.

Most RCTs showed no significant improvement in vitamin D supplementation except for one study, which reported significant improvement in cognition on taking vitamin D in Alzheimer's disease but was not taken much into consideration as it had a small sample size (n=210) and was for a shorter duration. Another study evidenced significant improvement in Mini-Mental State Examination (MMSE) score when memantine and vitamin D were taken together compared to when memantine and vitamin D were taken independently. Studies have shown that vitamin D deficiency is associated with an increased risk of developing cognitive impairment. But there is no sufficient evidence indicating vitamin D supplementation can improve cognitive function in Alzheimer's disease.

## Introduction and background

Among dementia in the elderly, Alzheimer's disease (AD) is one of the most common types of dementia. AD is a progressive neurodegenerative disorder causing cognitive impairment and memory loss with widespread cortical atrophy. Approximately 10% of persons over 70 years have significant memory loss, with more than 50% of the cause being AD [1]. The prevalence of AD increases with each decade, reaching 20%-40% of the population aged more than 85 years. AD is a multifactorial disorder due to a combination of age-related brain changes and genetic, environmental, lifestyle, vascular, and dietary risk factors. The pathogenesis of AD is thought mainly due to the accumulation of amyloid beta (A𝛽) 42 and tau protein in the form of neuritic plaques and neurofibrillary tangles, respectively. AD management is mostly symptomatic, and there is no curative or preventive treatment.

The effect of vitamin D on calcium and bone metabolism is well-known, but its effect on many chronic illnesses involving neurocognitive decline was recently noticed. Vitamin D acts through vitamin D receptor (VDR), a nuclear hormone receptor, which is present in neuronal and glial cells in almost all regions of the central nervous system (CNS). The areas essential for cognition are mainly expressed in the hippocampus, amygdala, hypothalamus, cortex, and subcortex [2].

Vitamin D helps in neuroprotection by regulating nerve growth and neurotrophic factors such as nerve growth factor, decreasing L-type calcium channel expression, and regulating the toxicity of reactive oxygen species and nitric oxide synthase [3-6]. Furthermore, vitamin D showed a lower accumulation of A𝛽 42 by enhancing its phagocytosis and amplifying brain-to-blood amyloid beta efflux transport at the blood-brain barrier (BBB), leading to fewer amyloid plaques [7-9]. Some studies showed an association between vitamin D receptor (VDR) gene polymorphisms and cognitive decline, AD [10,11]. Vitamin D deficiency has been linked with increasing hypertension, hyperlipidemia, myocardial Infarction (MI), and stroke, which are also risk factors for AD [12]. Vitamin D deficiency is more prevalent as age increases due to a decrease in cutaneous synthesis and decreased absorption of vitamin D. It is evident that low vitamin D concentration in older adults has shown an association with reduced cognitive performance and is more prevalent in those with AD [13,14]. This systematic review will study the efficacy of supplementing vitamin D on cognition in patients with early Alzheimer's disease.

## Review

Methods

**Figure 1:**
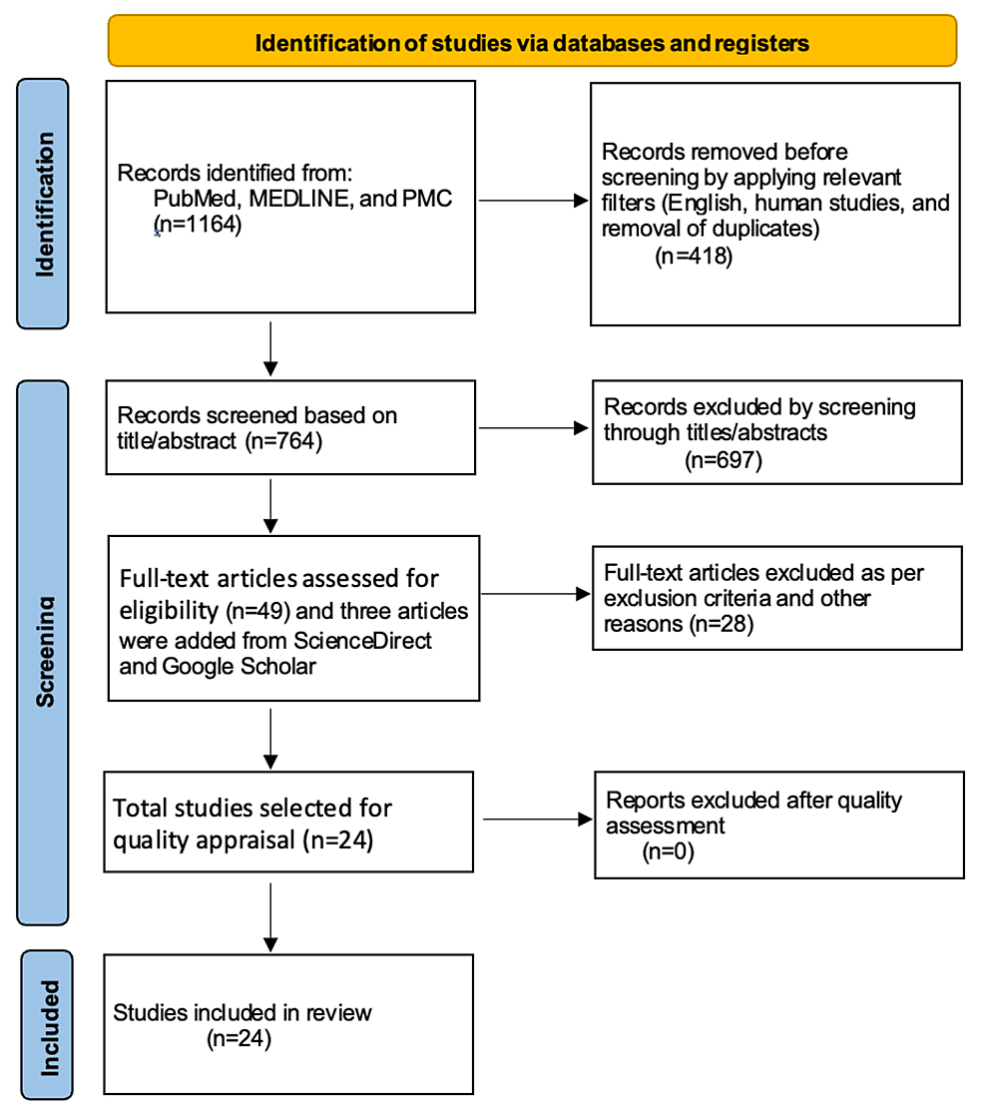
Preferred Reporting Items for Systematic Reviews and Meta-Analyses (PRISMA) flow diagram PMC: PubMed Central; n: number of articles; MEDLINE: Medical Literature Analysis and Retrieval System Online

Preferred Reporting Items for Systematic Reviews and Meta-Analyses (PRISMA) guidelines and principles were used to write this systematic review and report the results [15]. PRISMA flowchart is shown in Figure 1 [15].

Search Strategy

The major research literature databases and search engines such as Medical Literature Analysis and Retrieval System Online (MEDLINE), PubMed, PubMed Central (PMC), ScienceDirect, and Google Scholar were used to search appropriate keywords and Medical Subject Headings (MeSH) thesaurus and find relevant articles to the topic.

The ultimate MeSH strategy used for PubMed, PMC, and MEDLINE is as follows: ("Vitamin D/therapeutic use" {Majr} OR "Vitamin D/therapy" {Majr}) AND (“Alzheimer Disease/drug therapy" {Majr} OR "Alzheimer Disease/therapy" {Majr}) AND ("Cognitive Dysfunction/drug therapy" {Majr} OR "Cognitive Dysfunction/prevention and control" {Majr}). The keywords used for search in ScienceDirect and Google Scholar were vitamin D, Alzheimer's disease, and cognitive decline. These keywords were combined in several ways using the Boolean operators "AND," "OR," and "NOT" to locate relevant articles.

*Inclusion and Exclusion Criteria* 

We included articles published in English, focusing on the adult and geriatric population (≥40 years) and relevant to our research question. We excluded articles focusing on the population (<40 years), pregnant females, and unpublished or gray literature. A detailed description of inclusion and exclusion criteria is written in Table 1.

**Table 1:** A detailed description of the inclusion and exclusion criteria

Inclusion Criteria	Exclusion Criteria
1. Papers relevant to the question	1. Paper irrelevant to the question
2. Papers published in the English language	2. Papers published in languages other than English
3. Papers including adult (≥40 years) and geriatric population	3. Papers including the population (<40 years)
4. Papers with only full texts	4. Papers that are not full texts
5. Published papers	5. Papers of gray literature and unpublished articles

Analysis of Study Quality/Bias

Using standardized quality assessment tools, we critically evaluated selected studies, out of which 24 studies qualified as medium or high quality and were included in the review. The following tools were used for quality assessment: (a) Assessment of Multiple Systematic Reviews (AMSTAR) tool for systematic reviews and meta-analyses, (b) Newcastle-Ottawa scale for observational studies, (c) Scale for the Assessment of Narrative Review Articles (SANRA) checklist, and (d) randomized controlled trails (RCTs); Cochrane risk-of-bias assessment tool was used.

Data Extraction

Two investigators independently retrieved data and reviewed the eligible studies. The investigators would discuss the data for its relevance and design to eligibility criteria to reach an accord in case of disagreements. A third investigator was consulted for objectivity when the decision could not be made.

Results

In our initial search of MEDLINE, PubMed, and PubMed Central (PMC) databases, a total of 1164 articles were identified. Out of them, 418 articles were discarded after applying relevant filters per our eligibility criteria (human studies and English papers), and duplicates were removed. Then, the remaining articles (n=746) were screened based on titles, abstracts, full text, and detailed inclusion-exclusion criteria. We were left with 49 articles about our research question after the vigorous screening. Three articles were added by searching the relevant keywords to our topic in ScienceDirect and Google Scholar directly. A total of 24 studies were included for a thorough quality/bias assessment using standardized quality assessment tools and were included in this systematic review.

Of the 24 included studies, there were two observational studies, five randomized controlled trails (RCTs), one pilot study, and two meta-analyses, and the remaining were review articles. A total of 9592 participants were included in these studies. The summary characteristics of articles included in the study are in Table 2.

**Table 2:** Summary characteristics of studies included in the analysis of the role of vitamin D deficiency and its supplementation in cognition and Alzheimer's disease

Author and Year of Publication	Type of Study	Purpose of Study	Invention or Intervention Studied	Results/Discussions	Number of Patients
Schlögl and Holick, 2014 [12]	Review article	Association of vitamin D levels and neurocognitive function	Not available (N/A)	There is a higher chance of dementia or a significant decline in cognitive functions with vitamin D deficiency	Not available (N/A)
Annweiler et al., 2015 [16]	Review article, on the invitational summit	Relation between vitamin D and cognition in older people	Not available (N/A)	Concluded that vitamin D deficiency can increase the risk of cognitive decline and dementia	Not available (N/A)
Lu'o'ng and Nguyên, 2011 [17]	Review article	To know the role of vitamin D in patients with Alzheimer's disease	Not available (N/A)	Vitamin D improves cognitive function in Alzheimer's disease patients	Not available (N/A)
Aspell et al., 2018 [18]	Based on conference by the Nutrition Society Irish Section	The role of vitamin D in cognitive function as we age	Not available (N/A)	Vitamin D deficiency is seen most common in older people but unable to indicate optimal vitamin D levels to improve cognition in healthy elderly	Not available (N/A)
Keeney and Butterfiel, 2015 [19]	Accepted manuscript	Explained effects of vitamin D deficiency on the brain and its association with Alzheimer's disease along with the beneficial effects seen with vitamin D treatment	Not available (N/A)	Opined that vitamin D can be used as an adjunct therapy along with Alzheimer's disease therapy	Not available (N/A)
Annweiler, 2016 [20]	Review article	The neurological and clinical role of vitamin D on older adults, including implications for dementia onset and progression	Not available (N/A)	The correction of vitamin D deficiency in older people improves cognition. Association between vitamin D deficiency and cognitive disorders	Not available (N/A)
Balion et al., 2012 [21]	Systematic review meta-analysis	The association of vitamin D levels and cognition and dementia in adults	Not available (N/A)	Low levels of vitamin D is associated with increased risk of Alzheimer's disease and cognitive decline	Not available (N/A)
Brouwer-Brolsma and de Groot, 2015 [22]	Review article	The role of vitamin D in neurological functions	Not available (N/A)	Reported that calcifediol (25-hydroxy vitamin D) affects brain functions	Not available (N/A)
Soni et al., 2012 [23]	Review article	The association of vitamin D and cognition	Not available (N/A)	This study is about the association of vitamin D deficiency with cognition and dementia	Not available (N/A)
Pizza et al., 2020 [24]	Cross-sectional study	To know if vitamin D serum levels were associated with Alzheimer's disease patients from the Cilento area	Not available (N/A)	Vitamin D deficiency in patients from the Cilento region is independently associated with Alzheimer's disease	25
Bivona et al., 2021 [25]	Review article	Aims to determine if vitamin D can be used as a biomarker for diagnosis, prediction, and response to treatment in Alzheimer's disease and if vitamin D is a modifiable risk factor for Alzheimer's disease onset	Not available (N/A)	No clear evidence that vitamin D can be used as a biomarker to know the diagnosis and prognosis of Alzheimer's disease	Not available (N/A)
Sultan et al., 2020 [26]	Review article	The association of vitamin D deficiency with cognitive impairment and dementia	Not available (N/A)	Low vitamin D concentration is associated with cognitive impairment and dementia, although reverse causality is possible	Not available (N/A)
Banerjee et al., 2015 [27]	Review article	The effect of vitamin D on the progression of Alzheimer's disease and its potential as a therapeutic agent	Not available (N/A)	-	Not available (N/A)
Chai et al., 2019 [28]	A meta-analysis (research article)	Aims to find vitamin D deficiency associated with the risk of dementia and Alzheimer's disease	Not available (N/A)	Stronger association of severe vitamin D deficiency with both dementia and Alzheimer's disease compared to moderate vitamin D deficiency	Not available (N/A)
Landel et al., 2016 [29]	Review article	The effect of vitamin D levels on neurocognitive function	Not available (N/A)	Suggest the association of increased risk of developing Alzheimer's disease and dementia with hypovitaminosis D	Not available (N/A)
Littlejohns et al., 2014 [30]	Prospective cohort study	To know whether vitamin D deficiency is associated with an increased risk of incidence of all-cause of dementia and Alzheimer's disease	Not available (N/A)	The association of low serum vitamin D levels with increased frequency of dementia and Alzheimer's disease	1658
Anastasiou et al., 2014 [31]	Review article	The effects of vitamin D on the nervous system	Not available (N/A)	Vitamin D3 (cholecalciferol) has a role in neurological functions and established low levels of calcitriol in Alzheimer's disease patients and cognitively impaired patients	Not available (N/A)
Rossom et al., 2012 [32]	Randomized controlled trials (RCTs)	The effects of vitamin D and calcium on cognition in elderly females	400 international unit cholecalciferol (vitamin D3) and 1000 milligram calcium carbonate	There is no significant association in the incidence of dementia/minimal cognitive impairment (MCI) between groups	4143
Jia et al., 2019 [33]	Randomized controlled trail (RCT)	The effects of giving vitamin D on cognitive function	800 international units/day of vitamin D and placebo (starch granules)	Significant improvements in plasma amyloid beta 42 levels and a significant increase in full-scale intelligence quotient (IQ) during the follow-up period in the intervention group	210
Annweiler et al., 2012 [34]	Randomized controlled trail (RCT)	To determine whether patient treatment with memantine plus vitamin D is more effective in improving cognition among Alzheimer's disease patients than memantine or vitamin D used alone	Memantine and vitamin D	Treatment with memantine plus vitamin D showed improvement in Mini-Mental State Examination score compared to memantine (or) vitamin D alone	43
Bischoff-Ferrari et al., 2020 [35]	Randomized controlled trail (RCT)	To test whether vitamin D, omega-3s, and exercise alone/in combination improved six health outcomes among older adults	2000 international units/day of vitamin D3 (cholecalciferol), 1 gram/day of omega-3s, and strength training exercise program	There is no significant improvement in the Montreal Cognitive Assessment (MoCA), one of the main outcomes among the six outcomes	2157
Annweiler et al., 2012 [36]	Comparative study	To determine if the dietary intake of vitamin D can be a predictor of dementia onset	Not available (N/A)	Higher dietary intake of vitamin D is related to lower Alzheimer's disease risk	498
Annweiler et al., 2011 [37]	Randomized controlled trail (RCT)	To compare the effect of vitamin D3 (cholecalciferol) and memantine 200 milligrams/day after 24 weeks on cognitive performance in patients with moderate Alzheimer's disease and related dementia (ADRD)	Vitamin D (cholecalciferol) one drinking vial of 100000 international units every four weeks	Not available (N/A)	120
Llewellyn et al., 2010 [38]	Randomized controlled trail (RCT)	The association of vitamin D deficiency with an increased risk of cognitive decline	Not available (N/A)	Over six years, a significant association is seen between low vitamin D levels and cognitive decline	858

Discussion

Pathogenesis of Alzheimer's Disease

Alzheimer disease (AD) is characterized by progressive memory and cognitive impairment, which deteriorates to complete disability and death within three to nine years of diagnosis [39]. The pathological hallmark of AD includes the deposition of amyloid beta peptides outside the cells forming senile plaques (SP) and intracellular hyperphosphorylated tau proteins in the brain [40].

AD is multifactorial due to the complex interaction between genetic, aging process, environmental, and lifestyle factors that leads to neuronal degeneration. Environmental and lifestyle factors escalate the risk of developing AD through cerebrovascular damage [41]. Major interrelated networks leading to neuronal and synaptic degeneration in AD were amyloid beta accumulation, hyperphosphorylation of tau protein, metal dysregulation, oxidative stress, mitochondrial dysregulation, and inflammatory reaction [27]. Amyloid beta protein is formed following the consecutive hydrolysis of amyloid precursor protein (APP) [42]. In AD, amyloid beta (A𝛽) 40 and amyloid beta (A𝛽) 42 are major types of amyloid beta peptides. Among them, A𝛽 42 is more prevalent in neuritic plaques and has a higher predisposition to aggregate and form the characteristic toxic amyloid fibrils [27]. Regardless of the evidence, it is still unclear how amyloid deposition and "tauopathy" contribute to the complexity and heterogeneity of AD.

Vitamin D Metabolism

Vitamin D is a prohormone. Ergocalciferol (vitamin D2) is found mostly in food, whereas cholecalciferol (vitamin D3) is synthesized from 7-dehydrocholesterol in the human skin by the photochemical reaction of ultraviolet B rays [43]. Ergocalciferol (vitamin D2) and cholecalciferol (vitamin D3) forms must undergo two enzymatic hydroxylation reactions to become biologically active. The first hydroxylation of vitamin D occurs in the liver by 25-hydroxylase enzyme forming 25-hydroxy vitamin D (25-(OH)D) or calcidiol [44]. The second hydroxylation of 25-hydroxy vitamin D occurs in the kidney by one 𝛼-hydroxylase and converts it to 1,25-dihydroxy vitamin D (1,25(OH)2D) or calcitriol, which is a biologically active form [45]. Calcitriol level is regulated by a feedback mechanism and by stimulating parathyroid hormone, blood calcium concentration, and various cytokines [46]. Vitamin D acts through vitamin D receptors (VDR), which are expressed in most brain areas [2].

The Role of Vitamin D

Vitamin D has anti-inflammatory, antioxidant, and neuroprotective properties and a role in calcium and bone metabolism. In vitro studies show that vitamin D can promote the clearance of amyloid plaques by stimulating phagocytosis by macrophages and reducing neurodegeneration [47]. Thus, vitamin D supplementation may enhance the cognitive ability of elderly people and maintain neuronal health.

Calcitriol plays an important role in the differentiation and maturation of neurons by regulating the synthesis of neurotrophic agents such as nerve growth factor (NGF) and glial cell line-derived neurotrophic factor (GDNF) neurotrophin-3 [3]. These nerve growth factors are also required to maintain and regulate the septohippocampal pathway's normal functioning, involved in learning and memory. Vitamin D also regulates the genetic expression of various neurotransmitters in the brain, particularly in the hippocampus [48].

Few studies states that intraneuronal calcium homeostasis is regulated by vitamin D through an L-type voltage-sensitive calcium channel (LVSCC) along those targeted by A𝛽 [4]. Gezen-Ak et al. show that the silencing of VDR causes a rapid increase in L-type voltage-sensitive calcium channel alpha (α)-1C (LVSCC-A1C) expression, indicating that chronic deficiency of vitamin D renders the neurons in the brain vulnerable to neurodegeneration [49]. Therefore, treatment with vitamin D leads to the downregulation of LVSCC expression and channel density in the plasma membrane of the hippocampal neurons, which protects the neurons from calcium excitotoxicity [4].

The suppression of proinflammatory cytokines in the brain may be the mechanism of action of vitamin D for neuroprotection and increase brain-to-blood A𝛽 efflux across the blood-brain barrier (BBB), resulting in a decreased number of amyloid plaques [27]. Vitamin D has shown protection against acute glutamate exposure in cultured rat's cortical neurons through the upregulation of VDR expression and antioxidant effects [5].

Vitamin D Relation With Neurocognition and Alzheimer's Disease

Llewellyn et al. found that low vitamin D levels were associated with an increased risk of losing points on the Mini-Mental State Examination (MMSE) in the elderly over six years [38]. A meta-analysis by Balion et al. compared mean MMSE scores with levels of 25-hydroxy vitamin D (25(OH)D), where a higher-average MMSE score was observed in those participants with higher 25(OH)D concentrations [21]. Chai et al. found a significant positive association between vitamin D deficiency and dementia and AD risk and that the association is proportionate to the level of vitamin D deficiency [28]. In 2014, Littlejohns et al. conducted a prospective study over 5.6 years that reported an increased risk association between vitamin D deficiency and AD [30]. Another recent cross-sectional study by Pizza et al. reported no significant association between vitamin D deficiency and AD. But this study was performed on a small group of patients [24].

AD was predisposed to vitamin D deficiency due to feeding difficulties along with less intake of food rich in vitamin D and inadequate sun exposure. Long-term prospective studies have demonstrated a temporal link between vitamin D deficiency and cognitive disorders. Older people with lower vitamin D levels were more likely to experience executive dysfunction and general cognitive decline than those with normal or higher vitamin D levels [38].

Neurons ineffectively use vitamin D due to changes in the receptors VDR and 1,25 membrane-associated rapid response steroid-binding (1,25-MARRS), genes involved in its action, and vitamin D metabolism resulting in neurodegenerative changes. The association between AD and polymorphisms of VDR and megalin strongly supports this notion and, therefore, explains the neurotoxic effects of VDR and 1,25-MARRS suppression [27].

The Role of Vitamin D in AD Treatment

The purpose of drug treatment in AD is to improve cognitive ability and slow the progression of symptoms. Four drugs are currently approved by the Food and Drug Administration (FDA) for treating cognitive symptoms of AD. They are anticholinesterase agents (galantamine, rivastigmine, and donepezil) and memantine (act by preventing excitatory neuronal damage) [50].

Several strategies were proposed to halt the disease progression, such as decreasing the synthesis of A𝛽 42 by 𝛽-secretases or 𝛾-secretases inhibition, increasing the amyloid beta clearance by active or passive immunization from the brain or promoting it's enzymatic degradation and the activation of nonamyloidogenic processing of amyloid precursor protein (APP) through 𝛽-secretase action modulation, preventing the aggregation and fibrillization of A𝛽 42, inhibiting tau phosphorylation and antibodies against A𝛽, and reducing inflammation or oxidative stress or excitotoxicity [50]. But still, they are in the trial period, or some trials have shown non-promising results.

There is no adequate treatment for AD, despite different treatment strategies. As we have discussed above, vitamin D interacts through different mechanisms in preventing AD and cognitive decline, indicating that vitamin D can be a multitargeted therapeutic option. Randomized controlled trials (RCTs) are required to know the actual vitamin D effectiveness in improving cognitive function, but there are few current RCT studies. In older females, increased vitamin D dietary consumption was linked to a decreased chance of developing AD, according to Annweiler et al.'s seven-year follow-up research [36]. The first of these experiments, the AD-IDEA trial, which examined the efficacy of vitamin D in Alzheimer's disease and related dementia (ADRD) patients, was a randomized, placebo-controlled study [37].

An RCT study by Rossom et al. reported no significant difference in dementia even on vitamin D and calcium supplementation [32]. Another study by Jia et al. concluded that using vitamin D daily for 12 months improved cognitive function in AD patients [33]. Rossom et al.'s study was given importance as it had a larger sample size and longer follow-up period compared to Jia et al.'s study.

A six-month study trial by Annweiler et al. reported that combined vitamin D and memantine could improve cognitive function, as evidenced by an improved MMSE score. Still, these studies had limited populations and duration [34]. In 2020, an RCT by Bischoff-Ferrari et al. evaluated the impact of vitamin D supplements on the Montreal Cognitive Assessment (MoCA) in a three-year follow-up and concluded that vitamin D has no impact on cognitive function improvement [35].

Additionally, even though blood 25-hydroxy vitamin D concentrations are linked to these function-specific domains of cognition, none of this research evaluated executive or episodic memory as outcome measures [12]. The present argument against vitamin D supplementation is from a few numbers of clinical trials. So, to know the effectiveness of vitamin D supplements against placebo better in patients with AD, additional well-conducted RCTs are essentially needed at this time.

Limitations

The limitations of this study are having a smaller number of RCTs to examine the effectiveness of vitamin D alone in AD. The main issues of the existing RCTs were their small sample size, the lack of agreement over the dose, and age at which vitamin D supplements are to be given to prevent cognitive impairment. Therefore, there is a need for large double-blind, randomized controlled trials to assess the benefits of vitamin D supplementation in preventing and treating cognitive impairment.

## Conclusions

Many studies evidenced various functions of vitamin D throughout the central nervous system and the association of its deficiency with an increased risk of cognitive decline in older adults. There is a high risk of developing vitamin D deficiency in older people mainly due to decreased dietary intake and the cutaneous synthesis of vitamin D, thus suggesting its supplementation in the prevention and treatment of cognitive disorders and AD. But only a few randomized controlled trials (RCTs) indicate significant vitamin D improvement (cholecalciferol, vitamin D3). Some RCTs reported no significant improvement. However, there is no clear evidence that vitamin D supplementation can prevent AD onset and halt its progression. Given the inclusive scientific studies, it is too early to recommend a specific vitamin intake that delays or slows down cognitive decline. Hence, long-term, randomized, placebo-controlled trials need to assess the potential benefits of vitamin D addition in treating AD and dementia patients.
